# Is there an optimal basis to maximise optical information transfer?

**DOI:** 10.1038/srep22821

**Published:** 2016-03-15

**Authors:** Mingzhou Chen, Kishan Dholakia, Michael Mazilu

**Affiliations:** 1 SUPA, School of Physics and Astronomy, University of St. Andrews, KY16 9SS, UK

## Abstract

We establish the concept of the density of the optical degrees of freedom that may be applied to any photonics based system. As a key example of this versatile approach we explore information transfer using optical communication. We demonstrate both experimentally, theoretically and numerically that the use of a basis set with fields containing optical vortices does not increase the telecommunication capacity of an optical system.

One of the most fascinating and powerful questions in photonics is the maximum transfer of information between a source (transmitter) and a detector (receiver) within a finite optical system. A way to approach this is to consider the number of orthogonal (independent) measures required to represent the optical field^1^. An understanding of this question paves the way not only to determine the maximal information we may encode on a given field but also to understand the imaging resolution limit we may achieve.

In the last decade, research has focused on routes to maximise the information we may encode upon a light field^2^,^3^,^4^,^5^,^6^,^7^. A prime example has been the use of orbital angular momentum (OAM) which may take on any integer multiple of *ħ* in magnitude^8^. An alternative point of view is that non-zero OAM is associated with the presence of optical vortices, the number of which can be increased and used to encode information^9^. Using OAM to transmit information has the practical advantage to simplify the multiplexing/demultiplexing procedure^10^,^11^ and the presence of optical vortices presents a certain resilience to noise at the expense of needing specially designed optics such as vortex fibres^12^. Further, photons encoded with OAM might allow the generation of engineered qubits in multidimensional quantum information systems^13^,^14^ and can be used to achieve spatial mode entanglement^15^,^16^. In contrast, spin angular momentum (polarisation) is constrained to values of ±*ħ*. However, it is illuminating to explore if the OAM Laguerre-Gaussian (LG) basis set in Hilbert Space does in fact offer more information capacity^11^,^12^,^17^,^18^,^19^,^20^,^21^ compared to a basis set with Cartesian symmetry, namely the Hermite-Gaussian (HG) modes. For a fair and direct comparison, we consider that the information transfer route is constrained by the same given finite aperture, which is always the case in any real optical system.

Ultimately, the amount of information that can be encoded, transmitted and received in an optical system depends upon the optical degrees of freedom^22^ supported by it, i.e. the number of orthogonal (independent) fields able to be detected after their propagation through the optical system. Starting from thermodynamic arguments^23^ or Weyl’s law^24^ it is possible to show that the use of OAM does not change the total amount of information that can be transferred. Further, it is put forward that LG modes with OAM perform worse in free space or atmospheric communication because of the excessive crosstalk or a poor SNR^24^. With rigorous numerical simulations, even more recently, Zhao *et al.* pointed out the OAM multiplexing will not increase the capacity limits of a fibre optical communication channel when compared with conventional multiplexing methods^25^. Similarly, their simulations show that OAM multiplexing achieves even worse spatial efficiency and effective degrees of freedom than conventional spatial-mode multiplexing.

In this paper, we provide a theoretical description of the optical degrees of freedom (ODoF) and their density based on the optical eigenmodes approach^26^. We directly show that the density of ODoF does not depend on the basis set used to describe the field propagating through the optical system. Using a spatial light modulator, we experimentally create two sets of orthogonal modes corresponding to HG and LG beams respectively. The beams are used to probe the ODoF of the optical system considered. The measured density of ODoF, irrespective of the basis set used shows no difference, despite the presence of optical vortices and OAM in the LG basis set. These results are verified through numerical simulations.

## Theoretical Description

We define the ODoF within a finite detection area as the number of orthogonal fields that can be created in this area weighted by their respective intensities. In free space, HG and LG beams form two families of orthogonal modes when considered in the infinite transversal plane. However, in finite detection areas, these modes lose their orthogonality. The approach of optical eigenmodes^26^,^27^ offers a natural way to define an orthogonal basis taking into account the finite size of the system (propagation and detection). Additionally, each optical eigenmode in the detector area is associated with an eigenmode of unit power in the input plane of an optical system^26^. In this context, we note that the eigenvalues of the intensity operator in the detector plane correspond to the intensity received by the detector and as such their sum defines the absolute ODoF reaching the detector. It is also possible to introduce a relative number for the ODoF by normalising the eigenvalues with the highest one^27^. The difference between the two versions corresponds to either including the propagation through the optical system in the absolute case or disregarding the overall losses incurred during propagation (relative case). The absolute version is similar to the case of optical conductance^28^ except for the orthogonality and normalization requirements in the finite input plane.

More precisely, for any arbitrary optical system we can define a set *N* of orthogonal input modes *E*_*j*_(*x*, *y*) with *j* = 1 ... *N* each associated with the output mode *F*_*j*_(*x*, *y*). In the input plane, we have


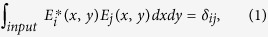


where * denotes the complex conjugate. In the output plane, we can define the local intensity for each illumination as 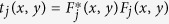 and the total transmitted intensity for illumination number *j* as


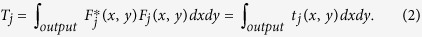


We remark that the output fields *F*_*j*_(*x*, *y*) are generally not orthogonal to each other due to various other restrictions in finite optical systems, such as finite size lenses and apertures. Using optical eigenmodes^27^ it is possible to define a set of illuminations that are orthogonal in both, the input and output planes


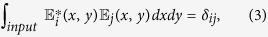



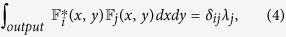


where 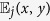 and 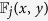 are the input and output optical eigenmodes for the considered optical system. Each of the 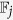 eigenmodes is associated with an eigenvalue *λ*_*j*_ corresponding to its intensity transmission coefficient. Due to their orthogonality, these optical eigenmodes can be regarded as independent degrees of freedom of the system and counting their number would result in estimating the ODoF that can be transmitted through the system. However, each mode has a different transmission efficiency determined by its eigenvalue, thus when counting the number of degrees of freedom *D* we need to weight each mode with its corresponding eigenvalue


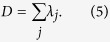


This definition is equal to the optical conductance definition^28^ provided that the illuminating beams considered are orthonormal, such as defined by *E*_*j*_(*x*, *y*). Indeed, we have


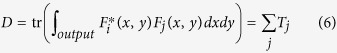


where tr(⋅) stands for the trace of 
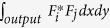
.

Furthermore, this property remains valid for any linear unitary transformation defined by matrix *U*_*ij*_ involving the input fields. In this case we have


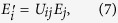



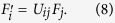


Implying the same total number of ODoF regardless of basis used, including the *OE*_*i*_ basis. Additionally, we can define the local density of ODoF as


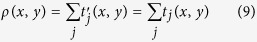


where 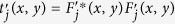. Using this definition, we have


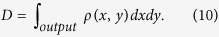


Introducing the ODoF this way ensures that the information capacity of an optical system is independent of the basis set used for as long as the basis set spans the same Hilbert sub-space i.e. it is complete with respect to optical systems. As a consequence to this definition, we note that the information capacity in any optical system is independent of any optical vortices embedded within a given field. We progress now to demonstrate this both experimentally and numerically using HG and LG beams.

## Results

### Orthogonality of HG/LG probing beams

To probe the ODoF in an optical system, we choose an orthogonal finite set of HG modes and an orthogonal finite set of LG modes as our probing beams. Both LG modes and HG modes form complete orthogonal sets of modes spanning the same Hilbert linear sub-space differing only in the presence, or absence of, optical vortices. Further, it is possible to introduce a unitary transformation matrix between the two basis sets. Indeed, any single LG mode can be decomposed into a set of HG modes and *vice versa*^29^,^30^. In this sense, these modes are very suitable as probing beams spanning the same Hilbert subspace, defined by the 36 probes used in our particular case.

**Figure 1:**
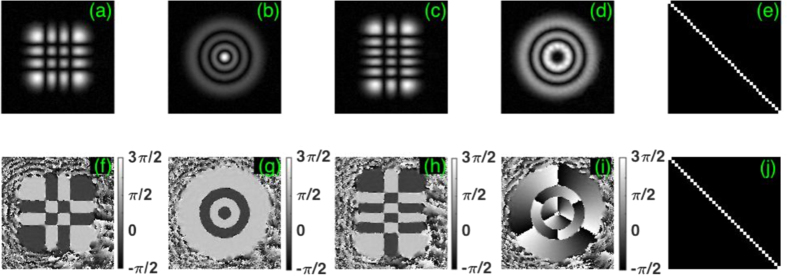
Measured complex fields of HG and LG probing beams on CCD_2_. (**a**)–(**d**) show the measured beam profiles of 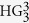, 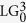, 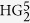 and 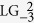 respectively (where the super- and subscripts of the HG beams relate to the horizontal and vertical index number and for the LG beams to the radial and azimuthal index number). (**f**)–(**i**) in bottom row show the corresponding measured phase functions. (**e,j**) Show the matrix of mode orthogonality in each set of 36 HG and LG probing beams respectively.

These beams are experimentally created using a spatial light modulator and the procedure for this is outlined in Supplementary Section 1. Using the iterating double-pinhole mode correction scheme, we achieve a high mode purity^31^ (in excess of 99%) for each probing beam while maintaining the orthogonality (error smaller than 0.14% as the maximum off-diagonal value shown in Fig. 1(e,j)) in each set of 36 probing beams. Figure 1(a–d,f–i) shows four experimentally measured complex fields corresponding to HG and LG modes after the double-pinhole corrections (see in Supplementary Section 1 for more details).

### Output beams

**Figure 2:**
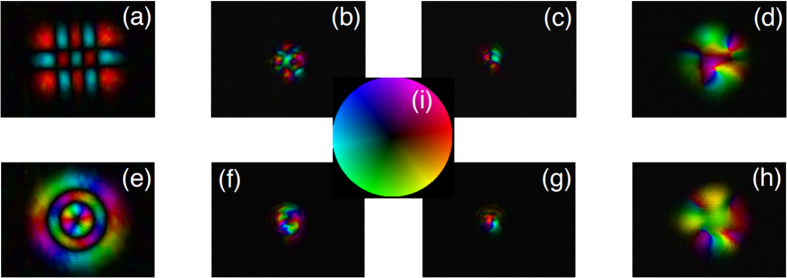
Measured output complex fields of probing beams, 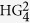 (top row) and 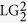 (bottom row), through (**a**,**e**) the free space, (**b**,**f**) a 5 *μ*m pinhole, (**c**,**g**) a 3 *μ*m pinhole and (**d**,**h**) a “few-mode” fibre. False colour map (**i**) is used to present the complex fields with the hue showing the intensity and the color showing the phase.

Figure 2 shows the measured output beams on CCD_1_ when 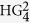 and 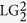 (mode index = 6) are used to probe the system, through which LG modes and HG modes will pass from a transmitter to a receiver. Each output beam is normalized by the total power of its corresponding input beam (see Supplementary Section 2)^32^. The measured complex fields contain similar profiles to the original incident probing beams in the free space case. However, a smaller number of modes are observed after the propagation through an optical restricting element such as a pinhole or a “few-mode” fibre (a single mode fibre used at the wavelength shorter than its operating wavelength, which supports a few modes^6^. The fibre used in our case allows less than 20 modes as indicated by the later experimental results).

### Density of ODoF

**Figure 3:**
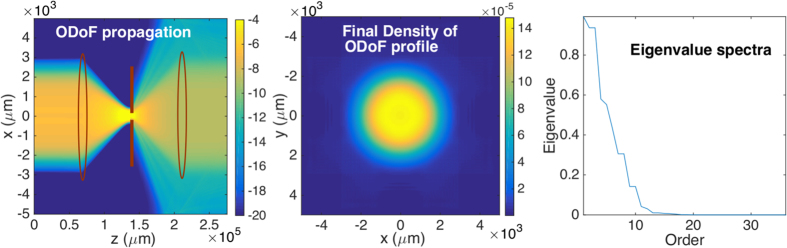
Numerical simulation of propagation of the ODoF. Logarithmic plot of the density in propagation plane (**a**) and in the output transversal plane (**b**). Plot of normalised eigenvalue spectra of the OEi of the system (**c**). Note that the degenerate eigenvalues originate from the time reversal invariance (see Supplementary Section 4 for a discussion).

The density of the optical degrees of freedom can be viewed as the information capacity of an optical system. In this context, we can numerically evaluate the density of ODoF in the absence or presence of vortices, i.e. when using HG or LG beams to probe the system. Figure 3(a,b) show the propagation of the density of ODoF through a system consisting of a two-lens telescope with a restricting aperture in the focal plane of the first lens. The observed density is identical in the HG and LG cases provided these probe the same Hilbert subspace (see the choice of the subspace considered in Supplementary Section 1). Same considerations deliver, in Fig. 3(c), identical eigenvalue spectra in both cases.

**Figure 4:**
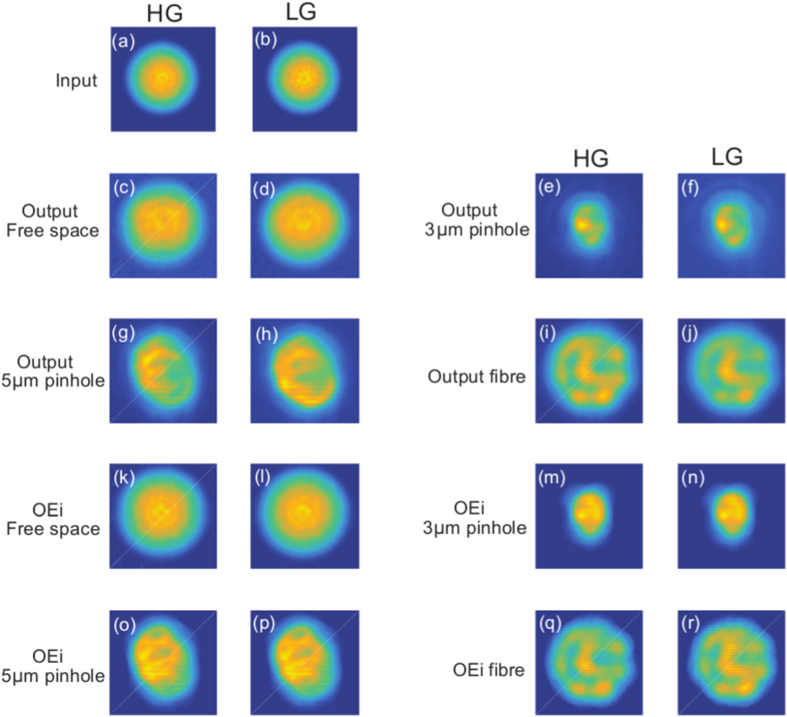
Total intensity of all input (**a**) measured HG probing beams and (**b**) measured LG probing beams. Total intensity of measured outputs of HG probing beams and LG probing beams through the free space (**c,d**), a 3 *μ*m pinhole (**e,f**), a 5 *μ*m pinhole (**g,h**), and a “few-mode” fibre (**i,j**). Total intensity of all eigenmodes of HG probing beams and LG probing beams through the free space (**k,l**), a 3 *μ*m pinhole (**m,n**), through a 5 *μ*m pinhole (**o,p**), and a “few-mode” fibre (**q,r**).

Experimentally this can be observed by confirming that the total intensity of all HG probing beams is exactly the same as the total intensity of all LG probing beams, as shown in Fig. 4(a,b) for all input HG/LG probing beams. This is valid for the total intensity of all output beams after propagation though the free space or any other optical restricting element (a 3 *μ*m pinhole, a 5 *μ*m pinhole and a “few-mode” fibre). Analogous arguments are valid when considering the total intensity using the measured optical eigenmodes of the system.

### Normalised eigenvalue spectra and ODoF

**Figure 5:**
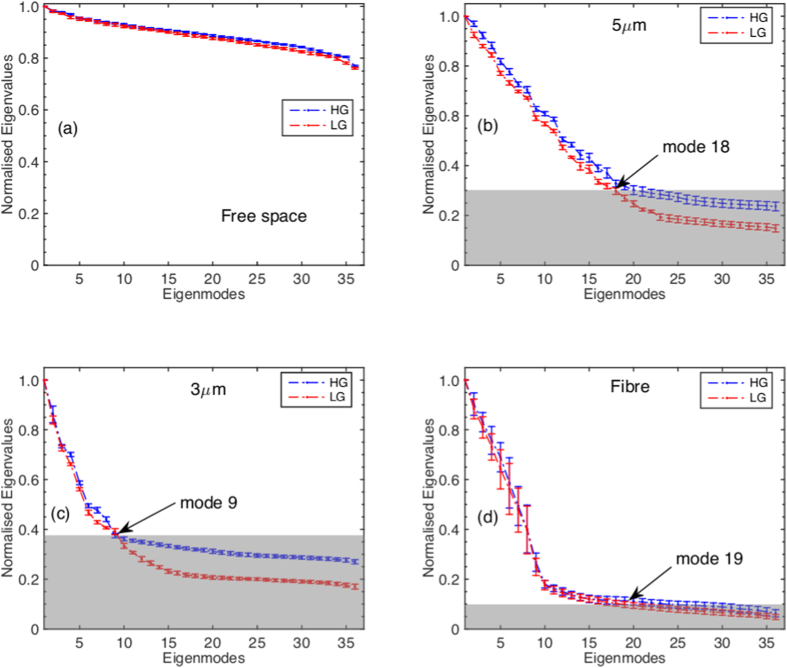
Eigenvalue spectrum for different optical restricting elements, (**a**) the free space, (**b**) a 5 *μ*m pinhole, (**c**) a 3 *μ* pinhole and (**d**) a “few-mode” fibre. Grayed-out areas show the noise levels (see Supplementary Section 5 for more discussions, code and data available online [34]).

Figure 5 shows the normalised eigenvalue spectra of the system with different optical restricting elements (see Supplementary Section 3 for the normalization factors). We observe that above the noise level (greyed-out areas), the eigenvalue spectra corresponding to LG and HG illumination have similar behaviour. This can also be verified by the measured total number of ODoF (*D*) as shown in Table 1.

## Discussions

The concept of ODoF in a linear optical system has been applied here in the context of information capacity. The results presented here show that the presence of the vortex does not enable any additional degrees of freedom to be accessed by the optical field. This is verified, theoretically (Fig. 3) and experimentally (Fig. 4). Indeed, the equivalence between the ODoF and the information capacity of an optical system ensures that the basis set used to carry information, with or without vortices, does not change the global capacity or the local density of information accessible. Figure 4 illustrates the case of the global capacity of different systems when illuminated by HG and LG beams. These results are summarised in Table 1. More precisely, we can state that this behaviour is reproduced locally i.e. the density of ODoF is invariant with respect to the presence or absence of vortices provided that the illuminations used probe the same linear sub-space. Together with the numerical simulations and theoretical definitions, we can conclude that the number of ODoF is not defined by the beams used to carry the information but by the system considered.

Ultimately, the concept of density of ODoF and number of ODoF is applicable to all linear optical systems and allows us to introduce theoretical limits to information capacity regardless of polarisation state or OAM of the light field used. Future work will look into extending this concept to the non-linear systems and the exploring the link between the density of ODoF and the imaging resolution limit.

## Methods

### Numerical simulation

The numerical simulations modelling the ODoF (Fig. 3) solve the paraxial equations using the split step approach implemented in Matlab. We consider a beam propagating through a pair of thin lenses modelled using a spherical phase mask and an aperture in the focal plane of the first lens modelled using an amplitude transmission mask. The lateral boundaries of the computational region are absorbing.

### Experimental set-up

**Figure 6:**
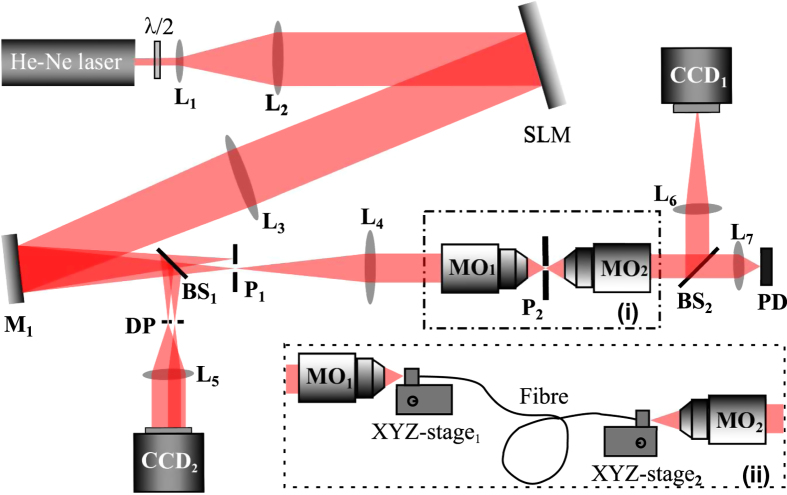
Schematic of the experimental setup. L_1_–L_7_ are lenses. P_1_ and P_2_ are pinholes. BS_1_ and BS_2_ are beam splitters. PD is a photon detector. DP is a double-pinhole. MO_1_ and MO_2_ are two identical microscope objectives.

An experimental setup, as shown in Fig. 6, is built to explore the number of ODoF in the system when different optical restricting elements and different probing beams are used. A collimated He-Ne Laser (*λ* = 632.8 nm, 5 mW) is expanded and projected onto the spatial light modulator (SLM, Holo-Eye HEO 1080p) which is used to create different probing beams. The first diffraction order is selected by a pinhole (P_1_) and then sent through an optical restricting element via a lens (L_4_) and a microscope objective (MO_1_) (Nikon, 20X/NA0.5/WD0.21 mm). Another identical microscope objective (MO_2_) is placed opposite to MO_1_. Both objectives share the same focal plane which is imaged onto the CCD plane (CCD_1_, Basler piA640-210 gm) with a lens (L_6_). Pinholes (P_2_) are placed on the common focal plane of those two microscope objectives as shown in the dash-dotted rectangle (i) in Fig. 6. When using the “few-mode” fibre, we need to modify our setup by replacing the pinhole with the fibre and two fibre stages as shown by the dash-line rectangle (ii) in Fig. 6. A photon detector (PD) is also used to monitor the output power from the beams collected. A beam splitter (BS_1_) is used to split a small part of the first and zero-th diffraction orders to realize the mode correction for all probing beams using a double-pinhole (DP), a Lens (L_5_) and a CCD (CCD_2_, SBIG STF-8300M).

### Optical restricting elements

The effective number of ODoF transmitted by an optical system is always finite because of noise^22^. Any system containing a restricting element, such as a pinhole or a “few-mode” fibre, will further limit the total number of transmitted modes allowing for the use of such a system to explore the effect of vortices on the ODoF. The optical restricting elements used in our experiment are pinholes (P_2_) (*ϕ* = 3 ± 0.5 *μ*m and *ϕ* = 5 ± 1 *μ*m), or a “few-mode” fibre (Thorlab P1-2000-FC-1, core radius = 5.91 *μ*m, L = 0.5 m).

### HG/LG probes preparation

In the absence of any restricting optical element, our setup allows for the propagation of modes up to mode index 7 (as defined in refs 29 and 30). Modes with higher mode index value are clipped by the CCD (CCD_1_). As a result, a set of 36 LG and 36 HG probing beams with their mode indices ranging from 0 to 7 are selected to explore the ODoF in our optical system. These two sets of modes are also completely convertible from one to another.

### Probing mechanism

Two sets of phase masks 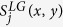 and 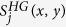, with *j* = 1 ··· 36, and where (*x*, *y*) are the coordinates on SLM plane, are used to create two orthogonal sets of HG/LG probing beams as our inputs. Each phase mask *S*_*j*_(*x*, *y*) will be co-encoded with a reference beam *S*_*r*_(*x*, *y*) onto the SLM^33^,


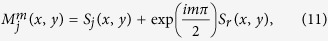


with *m* = 0, 1, 2, 3. Each of these masks creates a beam defined by the fields 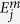. Similarly, the reference mask *S*_*r*_(*x*, *y*) creates a defocussed Gaussian beam with its beam waist large enough to cover all probes in order to interfere with all probing beams properly. The reference Gaussian beam is also mode corrected using the same double-pinhole correction scheme. Using the detected output fields 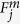, we can deduce the OEis and their eigenvalues^26^,^27^.

We probe the system multiple times with the same set of probing beams to avoid any systematic variance. The total ODoF for different optical restricting elements and different probing beams are calculated with a 2*σ* confidence derived from the integration of each eigenvalue spectrum above the noise level.

## Additional Information

**How to cite this article**: Chen, M. *et al.* Is there an optimal basis to maximise optical information transfer? *Sci. Rep.* **6**, 22821; doi: 10.1038/srep22821 (2016).

## Supplementary Material

Supplementary Information

## Figures and Tables

**Table 1:** Measured number of ODoF (±2*σ*).

Probes	Medium				
	Free space	5 *μm*	3 *μm*	Fibre	
HG	32.13 ± 0.19	11.58 ± 0.30	5.67 ± 0.14	8.99 ± 1.47	Relative ODoF
LG	31.78 ± 0.28	10.86 ± 0.07	5.48 ± 0.07	8.31 ± 1.68	
HG	34.72 ± 0.34	7.65 ± 0.79	3.25 ± 0.18	9.74 ± 1.66	Absolute ODoF
LG	33.64 ± 0.37	7.28 ± 0.69	3.12 ± 0.16	9.08 ± 1.89	
