# Supplementary material for: Is there an optimal basis to maximise optical information transfer?
Source: Sci Rep. 2016 Mar 15;6:22821. doi: 10.1038/srep22821 (PMC4791631; doi:10.1038/srep22821)
Supplement: Supplementary Information [file srep22821-s1.pdf]

Is there an optimal basis to maximise optical information transfer?

Mingzhou Chen, Kishan Dholakia and Michael Mazilu
School of Physics and Astronomy, University of St. Andrews, KY16 9SS, UK.

Supplementary information

1 Mode correction for HG/LG probing beams

Hermite-Gaussian (HG) modes and Laguerre-Gaussian (LG) modes can form a complete orthogonal set of modes. The mode index N of HG_m^n and LG_ℓ^p is defined as $N = m + n$, or $2p + |\ell|$. Each LG(HG) mode can be transformed from a set of corresponding HG(LG) modes with the conditions of $\ell = n - m$, $p = \min(n, m)$ [29, 30]. The mode purity can be calculated as an inner product of the required theoretical mode and the measured mode [29]. The orthogonality error is obtained from the maximum off-diagonal value in the orthogonal matrix of the whole set of measured probing modes.

To ensure the mode purity of each HG/LG probing beam and the orthogonality in each set of HG/LG probing beams created by SLM, we need to correct each mode with a double-pinhole correction scheme. As shown in Fig. 5, a beam splitter (BS_1) is used to divert part of light from the main optical axis onto a double-pinhole (DP). The diameter of the big pinhole has the same size as P_1 ($\phi = 4$ mm) which is used to selects the same first diffraction order only. The zero-th diffraction order is filtered through the other 20μm pinhole on the DP in order to obtain a reference beam with a planar wavefront after the lens (L_5). The separation distance between these two pinholes on DP is 4 mm which is determined by the grating used on SLM. The interference pattern between the first diffraction order and the reference beam is then recorded by a CCD (CCD_2, SBIG STF-8300M). With a well-known FFT fringe analysis method [S1], the complex field of the probing beam can be retrieved from the recorded fringe pattern. Because the probing beam and the reference beam are in the common path, we can obtain stable fringe patterns with high visibility, which in turn reduces the noise in the final calculated complex fields. The mode correction scheme is then can be described as following steps.

a. Encode a complex field $S_0(x, y) = u_0(x, y) \exp[i\phi(x, y)]$ of one LG/HG mode onto SLM with a complex-amplitude modulation method [31].

b. Record an interference pattern and calculate the complex field $S_c(u, v)$ on the CCD plane.

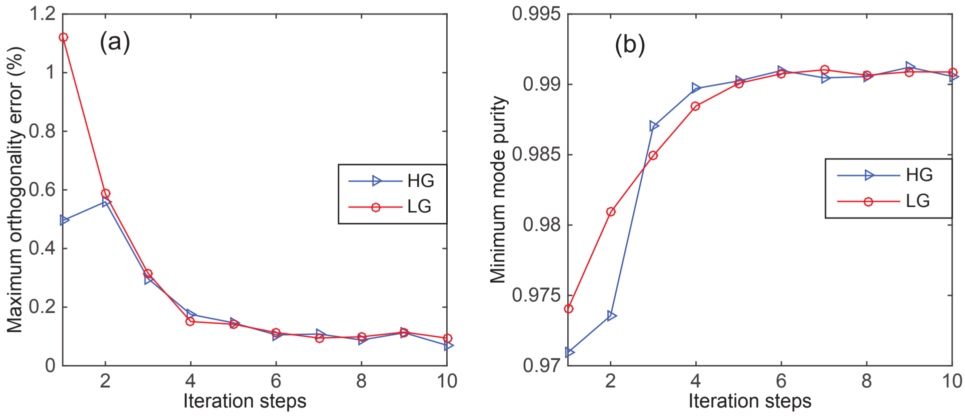

Figure S1: Plots of the maximum orthogonality error in each set of probing beams (a) and the minimum mode purity of all probing beams (b) as a function of iteration steps.

c. The measured complex field $S_c(u, v)$ can be transformed back onto the SLM plane as $S^{'}(x, y)$ with a precise image registration method [S2] since the CCD plane is on the image plane of SLM.

d. The mode purity is estimated from the inner production of the measured mode $S^{'}(x, y)$ and theoretical mode $S_0(x, y)$. Then locally correct the probing beams on the SLM if the mode purity is not acceptable. A new the complex field $S_0^{'}(x, y)$ can be calculated and then applied onto SLM.

e. Repeat steps (a) to (d) to correct all other probing beams.

f. Calculate the orthogonality error of the whole set of probing beams. Repeat step (a) to (e) to do further mode corrections until the orthogonality error is small enough.

With several iteration steps, a high mode purity can be achieved while maintaining the orthogonality in each set of probing beams, as shown in Fig. S1. With such a correction scheme, minimum mode purity can be finally achieved as high as 0.99 while maximum orthogonality error is as low as 0.14% in both sets of HG/LG probing beams.

2 Total power of input probing beams

The total power in each probing beam is measured by a photo diode (PD) when no optical restricting element is used. These 36 probing beams have the same optic axis, waist size and waist position, but different mode indices in order to keep the orthogonality. As shown in Fig. S2, the measured power

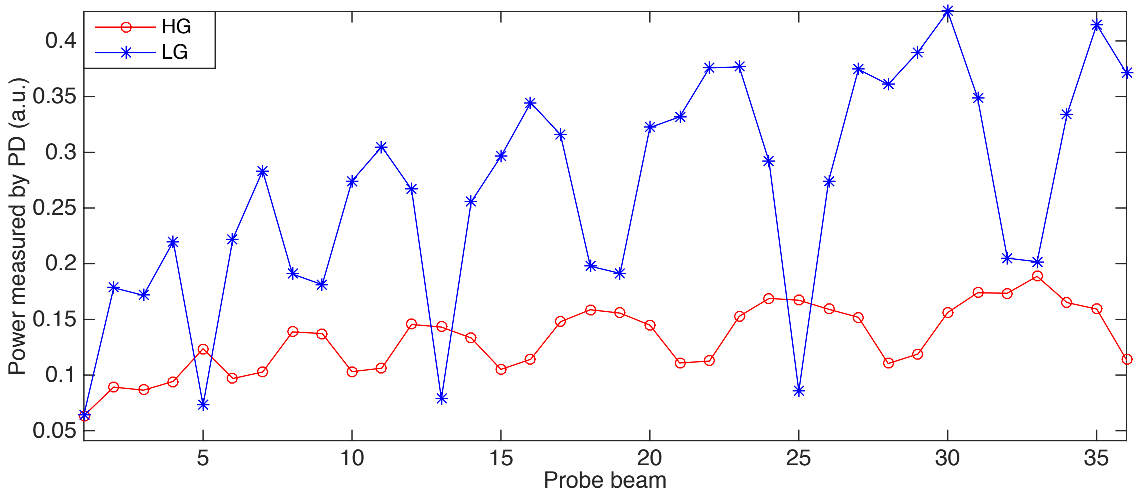

Figure S2: Power of 36 input HG/LG probing beams measured by PD.

Table S1: Normalization factors.

	Medium			
Probes	Free space	5μm	3μm	Fibre
HG	1.075±0.006	0.661±0.033	0.573±0.012	1.602±0.184
LG	1.083±0.006	0.671±0.031	0.569±0.013	1.612±0.182

in each probing beam varies a lot and is used to normalize each corresponding output beam [32].

3 ODoF normalization factors

As introduced in theoretical description section, we can have a relative number of ODoF if the eigenvalues are normalized by the highest one. Table S1 shows these normalization factors between absolute and relative number of ODoF.

4 Degenerate eigenmodes

In Figure 3 we observe that all eigenvalues are twice degenerate i.e. there are two optical eigenmodes associated with each distinct eigenvalue. Physically, these eigenvalues represent the coupling efficiency or transmission coefficient of the optical system between the input plane and the detector plane. The origin of the twice degenerate eigenmodes is the time reversal invariance of

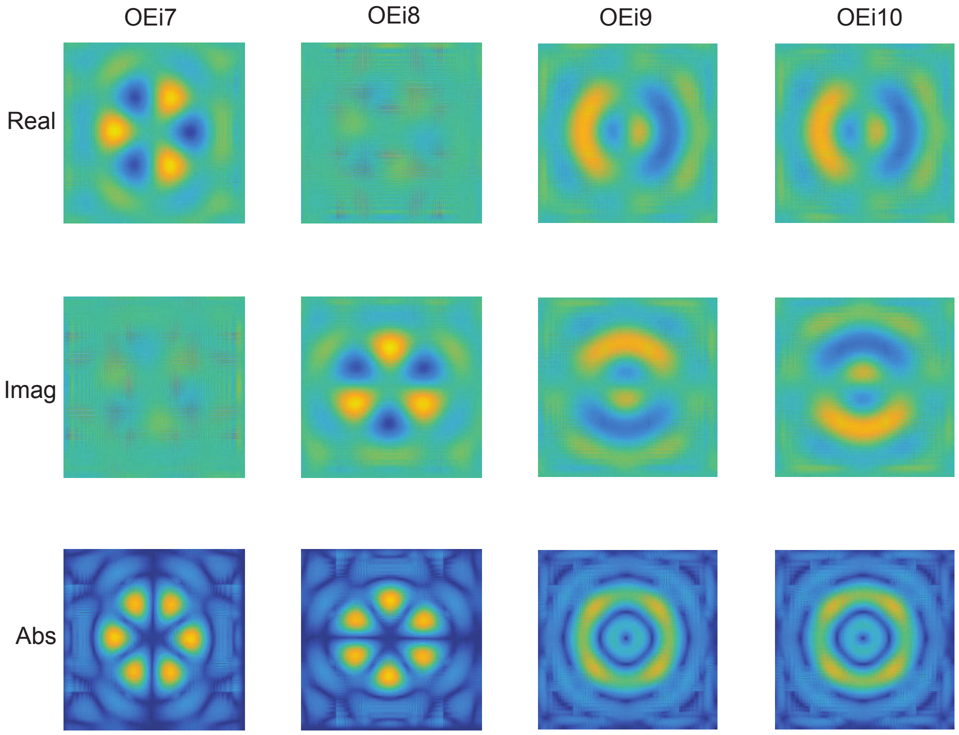

Figure S3: Eigenmodes in numerical simulations. Real, Imag and Abs denote taking the real part, the imaginary part and the absolute value of a complex field respectively.

the wave equations. Figure S3 shows the fields and intensity associated with four optical eigenmodes. The first two and last two are each having the same eigenvalue. We observe that using a superposition between the degenerate eigenmodes it is possible to define two modes that are complex conjugate to each other and orthonormal. However, taking the complex conjugate of a solution of the wave equation is equivalent to the time reversal which leaves the wave equation invariant.

5 Eigenvalues cut-off

From the measured optical eigenmodes, it can be observed that no signal can be detected for those high order eigenmodes. As shown in Fig. S4, all eigenmodes are detectable for the free space case while there are thresholds as 18, 9 and 19 eigenmode for 5μm pinhole, 3μm pinhole and a “few-mode”

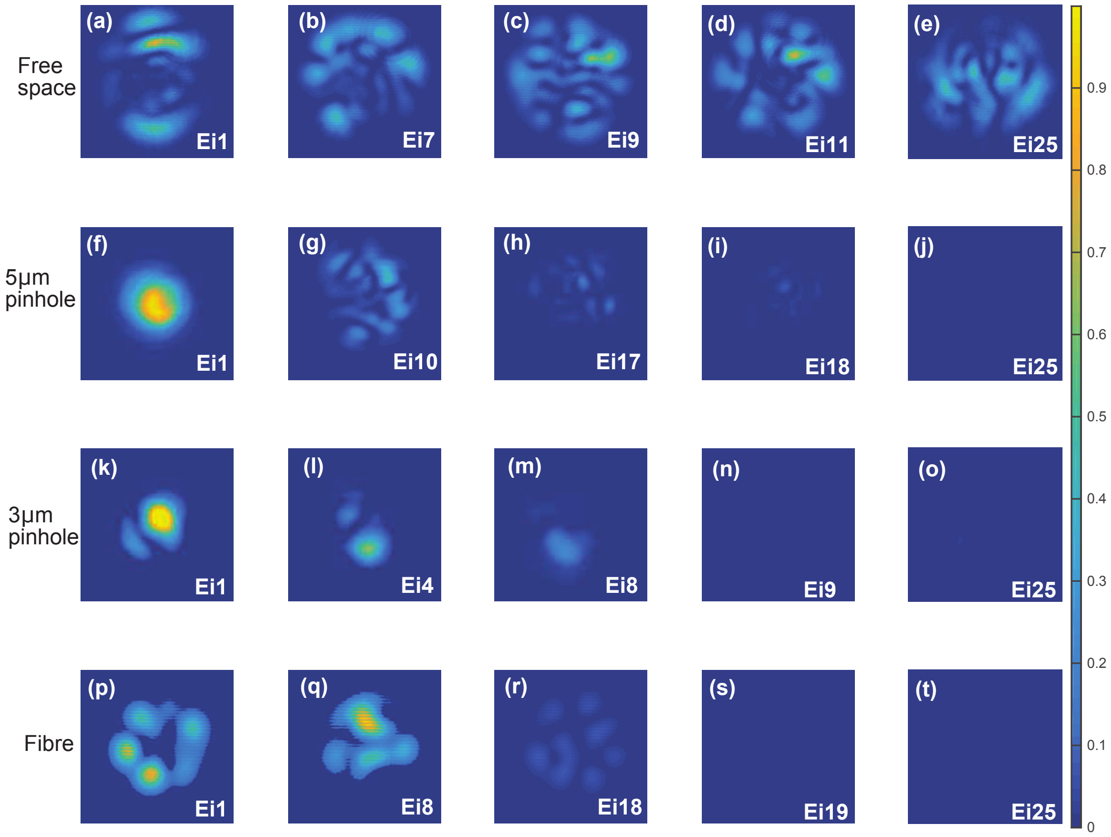

Figure S4: Measured optical eigenmodes through the free space, 5μm pinhole, 3μm pinhole and a "few-mode" fibre.

fibre respectively. Those eigenvalues corresponding to the eigenmodes order higher than the threshold will be considered as noise and will NOT be counted for the ODoF.

References

[S1] Takeda, M., Ina, H. & Kobayashi, S. Fourier-transform method of fringe-pattern analysis for computer-based topography and interferometry. *J. Opt. Soc. Am.* **72**, 156–160 (1982).

[S2] Thévenaz, P., Ruttimann, U. & Unser, M. A pyramid approach to subpixel registration based on intensity. *IEEE Trans. Image Process.* **7**, 27–41 (1998).
